# Determination of 3-MeO-PCP in human blood and urine in a fatal intoxication case, with a specific focus on metabolites identification

**DOI:** 10.1080/20961790.2021.1928821

**Published:** 2021-06-14

**Authors:** Nadia Arbouche, Pascal Kintz, Cecile Zagdoun, Laurie Gheddar, Jean-Sébastien Raul, Alice Ameline

**Affiliations:** a Institut de Médecine Légale, Strasbourg, France; b X-Pertise Consulting, Mittelhausbergen, France; c Unité Médico-Judiciaire, Hôpital Emile Muller, Mulhouse, France

**Keywords:** Forensic sciences, forensic toxicology, 3-MeO-PCP, metabolites, fatal intoxication, blood, urine

## Abstract

3-Methoxyphencyclidine (3-MeO-PCP) is a new psychoactive substance that belongs to the phencyclidines family, first identified in Europe in 2012. This drug presents a stronger binding to *N*-methyl-D-aspartate (NMDA) receptors when compared to phencyclidine, which results in more potent effects, even at low concentrations. Very few articles have been published regarding 3-MeO-PCP in forensic toxicology. In this paper, the authors present a fatal 3-MeO-PCP intoxication case. In addition to the detection of the parent drug, metabolites were investigated in urine and, for the first time in the scientific literature, in blood. 3-MeO-PCP and its metabolites were quantitated by liquid chromatography-tandem mass spectrometry system (LC-MS/MS). Identification was confirmed by liquid chromatography-high resolution mass spectrometry (LC-HRMS). 3-MeO-PCP tested positive in femoral blood (3 525 ng/mL) and urine (7 384 ng/mL). The femoral blood concentration was higher than the fatal concentrations range already reported in the literature (from 50 to 3 200 ng/mL). 3-MeO-PCP metabolites, including *O*-demethyl-3-MeO-PCP, piperidine-OH-3-MeO-PCP, *O*-demethyl-piperidine-di-OH-3-MeO-PCP and piperidine-di-OH-3-MeO-PCP, were detected in blood. In addition, two new metabolites, *O*-demethyl-piperidine-OH-3-MeO-PCP and *O*-demethyl-cyclohexyl-OH, were identified in both blood and urine. Unfortunately, due to the lack of reference material on the market, it was not possible to measure the concentration of these metabolites. However, the ratios between the metabolites and the parent drug were useful to estimate their analytical response and prevalence. At this time, considering the low ratios (<1) between metabolites and parent drug, metabolites testing does not seem useful to increase the detection window of the drug.

## Introduction

The new psychoactive substances (NPS) are a class of drugs of abuse, in pure or in preparations forms, which are not, for the most part, controlled by the Single Convention on Narcotic Drugs of 1961 (https://www.incb.org/documents/Narcotic-Drugs/1961-Convention/convention_1961_en.pdf) or by the Convention on Psychotropic Substances of 1971 (https://www.incb.org/documents/Psychotropics/conventions/convention_1971_en.pdf), thus representing a public health problem. These drugs are marketed as substances with similar effects to traditional drugs, such as cannabinoids, cocaine, opiates, and amphetamines or pharmaceuticals, such as benzodiazepines and anabolic steroids. The “novelty” is in the infinite possibilities of modifying the chemi­cal structure of these molecules and in the rapidity of appearance on the market, exceeding the capacities of many states and making quite impossible to impose an international control on the matter.

Problems related to public health are mainly due to the fact that purity and composition of these substances are unknown. Until December 2019, more than 950 substances have been reported to the United Nations Office on Drugs and Crime (UNODC) Early Warning Advisory (EWA).

**Figure 1.**
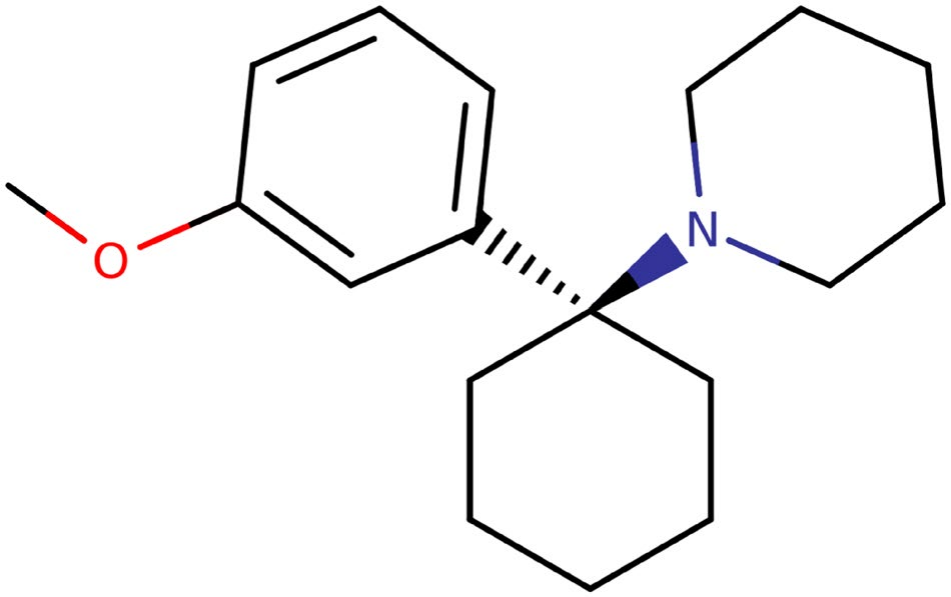
3-Methoxyphencyclidine (3-MeO-PCP) chemical structure.

3-Methoxyphencyclidine (3-MeO-PCP) (Figure 1) is a substance belonging to the family of phencyclidine (PCP) which is classified as arylcyclohexyla­mines. PCP was synthesized for the first time in 1950 and used as an anesthetic in the US until 1960 under the name of SernylTM. It was then withdrawn from the market due to its psychic effects, including psychosis, delirium and confusion. To escape national drug laws, phencyclidine structure has been continuously modified including modifications of the amino, phenyl, or piperidine rings [1]. According to the reports from the European Monitoring Center for Drugs and Drug Addiction (EMCDDA), 3-MeO-PCP was identified for the first time in Europe in 2012 [2]. PCP methoxylated analogues, such as 3-MeO-PCP and 4-MeO-PCP, were the initial mole­cules proposed as legal alternatives to PCP after its withdrawal from the market [3].

The most common routes of administration of 3-MeO-PCP are inhalation *via* snorting or smoking, oral administration, sub-lingual route and injection both intramuscularly and intravenously. Its activity threshold is reported to be 3–5 mg and its dissociative effect is obtained between 10 and 20 mg. The common dosage of abuse among users ranges from 3 to 20 mg [4].

3-MeO-PCP, like all PCP analogues, is an *N*-methyl-D-aspartate (NMDA) receptor antagonist, which is also the case of ketamine, diphenidine and methoxetamine. This pharmacological activity is the key of its anesthetic and dissociative effects. Since NMDA receptors are mainly involved in learning and memorization at the central nervous system level, symptoms such as amnesia, hallucinations and perceptual alterations may appear when NMDA activity is inhibited. The major effects described by the consumers are euphoria, dissociation and hallucinations. According to recent studies, 3-MeO-PCP seems to have a greater affinity for the binding site when compared to PCP for NMDA receptors and therefore a greater antagonist and inhibitory activity [5].

The identification of 3-MeO-PCP in non-fatal and fatal intoxication cases has been poorly described in the literature and the metabolism of 3-MeO-PCP has been seldom described in two papers [6,7]. Michely et al. [6] established the metabolism of 3-MeO-PCP using human liver microsomes and subsequent comparison with urine of rat after administration. However, this study was not applied to humans. Ameline et al. [7] also investigated the metabolism of 3-MeO-PCP using human liver microsomes and applied the findings to urine collected from two abusers. No one has reported metabolite(s) identification in blood.

In this paper, the authors present a case of fatal intoxication involving 3-MeO-PCP. This is the opportunity to highlight the laboratory strategy when dealing with NPS present on the market with positional isomers. First, the identification of 3-MeO-PCP and its discrimination from its isomer 4-MeO-PCP was performed using gas chromatography coupled to mass spectrometry (GC-MS) as it is not possible to do it by liquid chromatography since the two molecules have the same retention time and the same ion transitions. It is very important to discriminate the two isomers, as their toxicities are very different. In particular, 3-MeO-PCP has a stronger binding to NMDA receptors when compared to 4-MeO-PCP, which produces toxicity even at low concentrations. Once confirmed, quantification of 3-MeO-PCP in blood and urine was performed using liquid chromatography coupled to tandem mass spectrometry (LC-MS/MS). Finally, the authors focused their investigation to the identification of metabolites by liquid chromatography coupled to high resolution mass spectrometry (LC-HRMS) in both blood and urine.

## Case report

**Figure 2.**
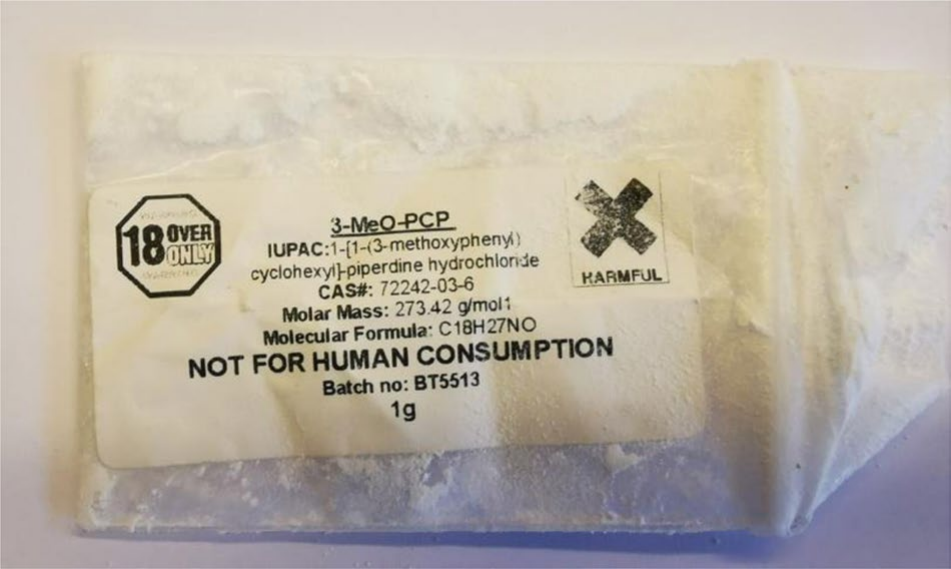
Plastic bag containing 3-methoxyphencyclidine (3-MeO-PCP) powder.

A 44-year-old male was found dead by his father. He was naked and lying on the ground on the outside terrace of his home. Some clothes and a “smiley” drawn were found on the ground. In the dining room of the deceased subject, the police found a plastic bag of white powder (Figure 2) with a label “3-MeO-PCP NOT FOR HUMAN CONSUMPTION”, several boxes of methadone capsules and a white powder deposited on a CD. Police investigations revealed that the subject was a drug addict since several years (cannabis and alcohol) and that he was treated in an addiction center with methadone.

During the external examination of the body, the medical examiner observed marks of cyanosis on the finger, toenails, lips and ears. The presence of foam in the nose was compatible with asphyxia syndrome and the presence of skin lesions in the elbows and knees was in accordance with a fall on these points. Since the signs of asphyxia were not considered specific to a cause of death, the pathologist suggested to the local prosecutors to request toxicological analyses.

Several biological and non-biological specimens were collected and placed under seal: femoral blood (collected without preservative), urine, the bag containing the white powder and the CD. These items were sent to the laboratory. Blood and urine were kept at +4 °C until analysis. The bag of powder and the CD were stored at room temperature. The pro­secutor did not request a full autopsy. Hair specimens were not collected, as the subject was quite bald.

## Material and methods

### Standard and reagents

Certified solution of 3-MeO-PCP, at 1 mg/mL in methanol, used to prepare the working solutions for the calibration curve was purchased from Sigma Aldrich (Saint-Quentin-Fallavier, France). MDMA-d5, used as internal standard, was obtained from Lipomed (Arlersheim, Switzerland) and diluted to appropriate concentrations. Ammonium formate, **>**99%, di-sodium tetraborate decahydrate and ammonium acetate were provided by VWR Chemicals (Leuven, Belgium). Formic acid for LC-MS was obtained from Carlo Erba (Chaussée du Vexin). HPLC-grade acetonitrile, methanol, dichloromet­hane, n-hexane, ethyl acetate and diethyl ether, acetic acid and 36% hydrochloric acid were obtained from VWR Prolabo (Fontenay-sous-Bois, France). Isoamyl alcohol, β-glucuronidase and heptafluorobutyric acid (HFBA) were purchased from Sigma Aldrich.

### 3-MeO-PCP analysis

#### Powder analysis

In order to firmly identify 3-MeO-PCP in all speci­mens and to establish the degree of purity in the powders deposited on the CD and contained in the plastic bag, GC-MS and LC-MS/MS were used. After scraping the dust deposited on the CD with a swab soaked in methanol and after correct dilution in methanol of the powder contained in the bag, the methanol solutions were evaporated to dryness and derivatized with 100 μL of HFBA at 60 °C for 30 min. After evaporation, the residue was reconstituted in 25 μL of ethyl acetate and injected in the GC/MSsystem.

LC-MS/MS allowed establishing the degree of purity by comparing a certified standard solution with the solution obtained from the powder seized at the same concentration.

#### Blood and urine analysis

Again, the biological specimens were tested by GC/MS to discriminate 3-MeO-PCP from 4-MeO-PCP. Briefly, 1 mL of blood and hydrolyzed urine were extracted with 1 mL of tampon borate buffer pH 9.5 and 5 mL of ethyl acetate in the presence of 100 ng of MDMA-d5, used as internal standard. After agitation and centrifu­gation, the supernatant was evaporated to dryness and the residue derivatized in presence of 100 μL of HFBA at 60 °C for 30 min. The derivatized sample was evaporated to dryness and reconstituted with 25 μL of ethyl acetate. Then 1 μL of derivatized sample was injected in the GC-MS system.

In order to identify and quantify 3-MeO-PCP by an LC-MS/MS system, a previously described and validated method was used to test for 3-MeO-PCP in blood and urine [7]. Briefly, 1 mL of specimen (blood and hydrolyzed urine) was subjected to an alkaline liquid-liquid extraction in presence of 2 ng of MDMA-d5. Subsequently, the organic supernatant was evaporated to dryness and reconstituted with 30 μL of 5 mmol/L ammonium formate buffer adjusted at pH 3 and transferred to injection vials for LC-MS/MS analysis.

A 6-point calibration curve was prepared using the following concentrations: 0.01, 0.1, 0.5, 1, 5, 10 mg/L of 3-MeO-PCP in blood and urine.

#### GC-MS conditions

One μL of the derivatized sample was injected in the GC-MS system. GC-MS analysis was performed on a PerkinElmer system (Clarus 680 & Clarus SQ 8 T; Waltham, MA, USA) using a PerkinElmer 30 m × 0.25 mm, 0.25 μm 5MS column for separation. The two isomers were differentiated by their retention time, 6.86 min for 3-MeO-PCP and 6.94 min for 4-MeO-PCP, while both have the same pattern of fragmentation (*m*/*z* 230, 272, 273, 121, 216).

#### LC-MS/MS conditions

LC separation was achieved using a Waters Acquity HSS C18 column (150 μm × 2.1 μm × 1.8 μm) with a controlled temperature maintained at 50 °C. A 2 μL injection with a 0.4 mL/min flow of formate buffer adjusted to pH 3 and 0.1% formic acid in acetonitrile was used. The gradient elution was as follows: the initial 13% B was increased to 50% over 10 min, 50% to 95% over 2.25 min and returned to initial conditions over 2.25 min. The total run time was 15 min and 3-MeO-PCP was eluted at 6.29 min.

A Xevo TQD triple-quadrupole mass spectro­meter (Waters Corporation, Milford, MA, USA) provided with a Z-spray electrospray ionization source (ESI) used in the positive ionization mode (ESI+) was used for analysis of the compound. The following condition were found to be optimal for the analysis of 3-MeO-PCP: capillary voltage at 1.5 kV; source block temperature at 150 °C; desolvation gas nitrogen heated at 600 °C and delivered at a flow rate of 1 000 L/h. The cone voltage and collision energy were adjusted to maximize the intensity of the protonated ion and to optimize the signal of the two most abundant ion products of 3-MeO-PCP: *m*/*z* 274 > 86.1 and *m*/*z* 274 > 189.1, and for the internal standard MDMA-d5 198.9 > 164.9. Transition 274 > 86.1 was used for quantification of 3-MeO-PCP. MassLynx 4.1 software (https://www.waters.com/webassets/cms/support/docs/71500113203ra.pdf) was used for quantification.

The LC-MS/MS instrument conditions were the same for blood and urine.

#### LC-HRMS conditions

In order to investigate the production of 3-MeO-PCP metabolites in blood and urine, an LC-HRMS system was used. Chromatographic separation was performed with the same equipment and parameters of the ones of the LC-MS/MS system. Detection was performed using a high resolution (XEVO G2XS Q-TOF, Waters Corporation) mass spectrometer operating in positive ion mode and in sensitivity mode. Desolvatation gas flow was set to 1 000 L/h at a temperature of 600 °C, the cone gas to 50 L/h and the source temperature set to 120 °C. The capi­llary voltage and the cone voltage were set to 0.5 and 20 V, respectively. In MS scanning, data were acquired from 50 to 1 000 *m*/*z*. Collision energy ranged from 10 to 40 V. UNIFI software (Ubiquiti Inc., New York, NY, USA) was used for data, chromatograms and spectra acquisition. UNIFI was also used to predict and to match potential metabolites.

## Results and discussion

For the identification of the white powder and the drug present in blood and urine, GC-MS was applied. Gas chromatography analysis allowed discriminating 3-MeO-PCP from its positional isomer 4-MeO-PCP, based on their respective retention times. The powder inside the plastic bag and the powder deposited on the CD were identified as 3-MeO-PCP. Unfortunately, liquid chromatography does not allow discrimination between the various positional isomers, since the retention time is identical as well as their fragmentation pattern. The discrimination of these two isomers is of importance as these molecules do not share the same toxicity. The degree of purity of the powder inside the plastic bag was measured at 86.2%.

3-MeO-PCP was quantified by LC-MS/MS in femoral blood and urine at the following concentrations: 3 525 ng/mL and 7 384 ng/mL, respectively. Additional tests revealed the presence methadone (94 ng/mL) and its metabolite, 2-ethylidene-1,5-dimethyl-3,3-diphenylpyrrolidine (EDDP, 16 ng/mL) at therapeutic levels. Recent cannabis use was also identified by the presence of Δ^9^-tetrahydrocannabinol (THC, 0.6 ng/mL) and its metabolite, THC-COOH (8.6 ng/mL).

The 3-MeO-PCP femoral blood concentration was higher than the highest one reported in the literature (50–3 200 ng/mL) [3,7–10]. In this case, given the very high drug concentration, the pathologist attributed the death to 3-MeO-PCP.

The distinction between concentrations from non-fatal (49–350 ng/mL)[10–12] and fatal intoxications (50–3 200 ng/mL) is not very obvious. Often, when concomitant use of other psychoactive substances occurs, the fatal concentrations of 3-MeO-PCP appear to be lower than the ones measured in single intoxications. The highest 3-MeO-PCP blood concentration (3 200 ng/mL) was reported by Mitchell-Mata et al. [3], who described a case of 3-MeO-PCP overdose in the presence of polysubstance abuse (methamphetamine). Johansson et al. [10] described a case where the only substance that contributed to death was 3-MeO-PCP measured at 380 ng/mL and a case where polydrug abuse caused the death with a 3-MeO-PCP concentration at 50 ng/mL, which is the lowest fatal concentration reported in the scientific literature. Bertol et al. [11] reported 56 non-fatal intoxication cases involving 3-MeO-PCP with concentrations ranging from 1 to 242 ng/mL, while De Jong et al. [8] reported a concentration of 152 ng/mL in a death due to 3-MeO-PCP. Bakota et al. [9] reported a case which death was attributed to a combination of 3-MeO-PCP (139 ng/mL) and other drugs of abuse (amphetamine and diphenhydramine).

There are very limited data dealing with 3-MeO-PCP metabolites characterization. Only two studies have been published. In 2017, Michely et al. [6] evaluated phase I and II metabolites of 3-MeO-PCP *in vitro*, and found more than 35 products. In 2019, Ameline et al. [7] identified some 3-MeO-PCP metabolites in the urine collected from two abusers.

To complete the data, the production of 3-MeO-PCP metabolites was targeted in both blood and urine samples of the deceased subject using an LC-HRMS system in the full scan mode. The major metabolite was *O-*demethyl-3-MeO-PCP (C_17_H_26_NO; *m/z* [M + H^+^] 260.2012) formed by the *O*-demethylation of the cyclohexyl ring of 3-MeO-PCP. The second largest metabolite identified comes from the mono-hydroxylation of the piperidine fragment, which is the piperidine-HO-3-MeO-PCP (C_18_H_28_NO_2_; *m/z* [M + H^+^] 290.2119). From the combination of the process of *O*-demethylation and hydroxylation derives the metabolites *O*-demethyl-piperidine-di-HO-3-MeO-PCP (C_17_H_26_NO_3_; *m/z* [M + H^+^] 292.2276) and piperidine-di-HO-3-MeO-PCP (C_18_H_28_NO_3_; *m/z* [M + H^+^] 306.2066).

**Figure 3.**
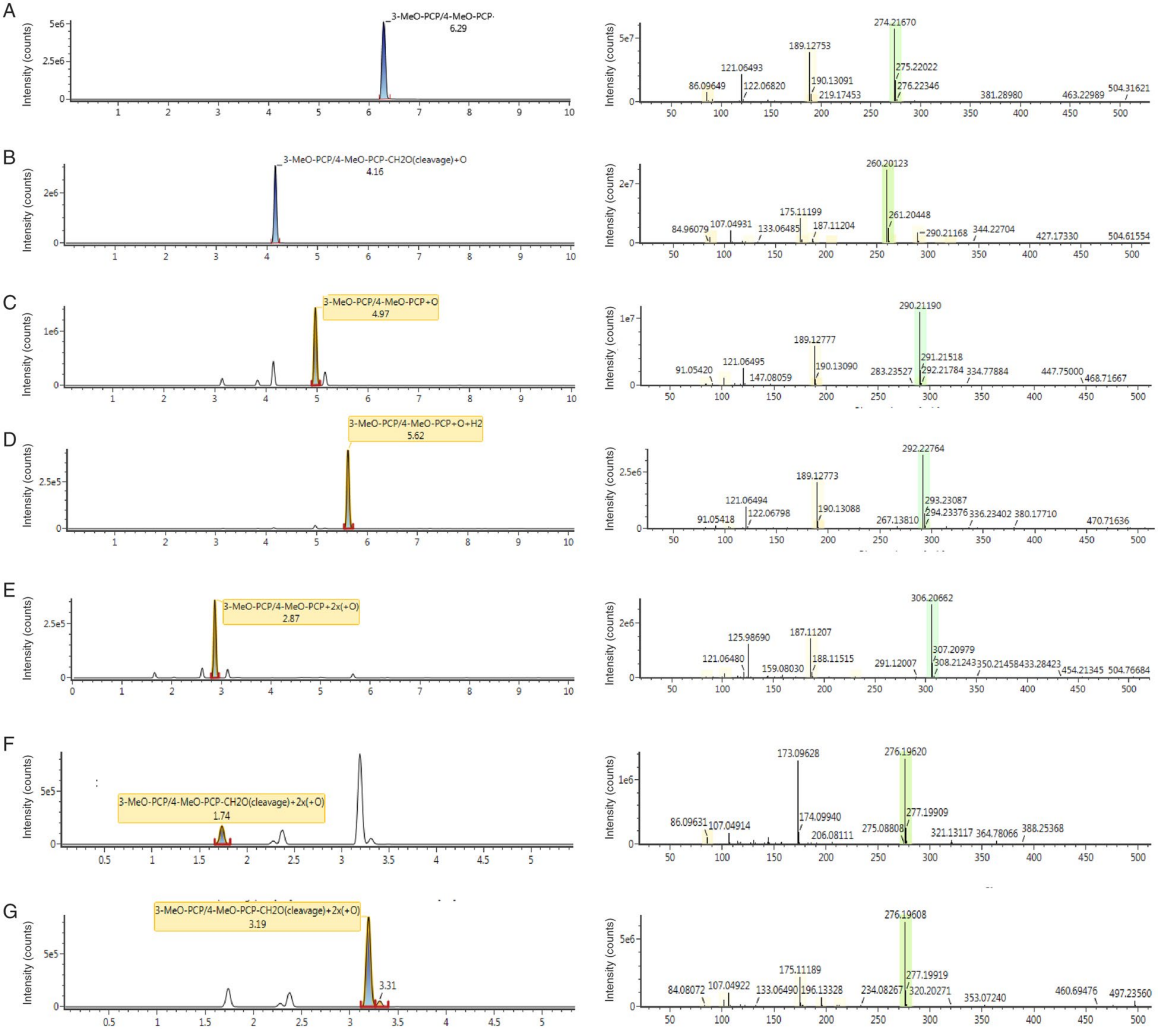
Spectral data information for 3-MeO-PCP, and metabolites found in urine of the fatal case, by liquid ­chromatography coupled to high resolution mass spectrometry (LC-HRMS). (A) 3-MeO-PCP (C_18_H_27_NO_2_; *m*/*z* [M + H^+^] 274.2167), (B) *O-*demethyl-3-MeO-PCP (C_17_H_26_NO; *m*/*z* [M + H^+^] 260.2012), (C) piperidine-HO-3-MeO-PCP (C_18_H_28_NO_2_; *m*/*z* [M + H^+^] 290.2119), (D) *O*-demethyl-piperidine-di-HO-3-MeO-PCP (C_17_H_26_NO_3_; *m*/*z* [M + H^+^] 292.2276, (E) piperidine-di-HO-3-MeO-PCP (C_18_H_28_NO_3_; *m*/*z* [M + H^+^] 306.2066), (F) *O*-demethyl-piperidine-HO-3-MeO-PCP (C_17_H_26_NO_2_; *m*/*z* [M + H^+^] 276.1962) and (G) *O-*demethyl-cyclohexyl-HO-3-MeO-PCP (C_17_H_26_NO_2_; *m*/*z* [M + H^+^] 276.1961).

In addition to the metabolites already described by Ameline et al. [7], two other metabolites were identified during these investigations: *O*-demethyl-piperidine-OH-3-MeO-PCP (C_17_H_26_NO_2_; *m/z* [M + H^+^] 276.1962) and *O-*demethyl-cyclohexyl-HO-3-MeO-PCP (C_17_H_26_NO_2_; *m/z* [M + H^+^] 276.1961). Chromatographic information and mass spectrum of the targeted metabolites are shown in Figure 3.

Unfortunately, it was not possible to quantify these 3-MeO-PCP metabolites as the reference material is not commercially available. However, it was possible to calculate the ratios between metabolites and 3-MeO-PCP using the areas in the full spectrum mode in both blood and urine (Table 1). These ratios were always lower than 1, confirming the initial report in urine published in the literature [7]. The major urinary metabolite was always *O-*demethyl-3-MeO-PCP which was at 42%, in our case. However, we cannot compare the results of our metabolites investigation in blood since it has never been described in literature.

**Table 1.** The ratio (peak area) between 3-methoxyphencyclidine (3-MeO-PCP) detected in blood and urine (525 and 384 ng/mL respectively, 100%) and its metabolites detected.

	Ratio (%)	
Metabolites	Blood	Urine
*O*-demethyl-3-MeO-PCP	17.0	42.0
Piperidine-HO-3-MeO-PCP	8.4	19.0
*O*-demethyl-piperidine-di-HO-3-MeO-PCP	3.3	7.0
Piperidine-di-HO-3-MeO-PCP	0.1	5.9
*O*-demethyl-piperidine-HO-3-MeO-PCP	0.9	13.0
*O*-demethyl-cyclohexyl-HO-3-MeO-PCP	0.1	2.3

Therefore, the identification of metabolites does not seem to increase the detection window of 3-MeO-PCP in either blood or urine. Since no controlled study on the pharmacokinetic of 3-MeO-PCP has ever been performed, it is not possible to certify that one of the metabolites does not increase the detection window of a consumption of 3-MeO-PCP at a given point of elimination. It is unknown if any of these metabolites would be detectable when 3-MeO-PCP is completely cleared from the body.

Some authors have tried to give some more information about the elimination kinetic of 3-MeO-PCP. For example, Johansson et al. [10] estimated that 3-MeO-PCP has an elimination half-life of about 11 h, after having analyzed four blood specimens collected from a 3-MeO-PCP intoxicated case at different times. This is in agreement with what was described by Backberg et al. [13] that suggested that 3-MeO-PCP may have an elimination half-life of about 10 h.

Despite the absence of suitable literature, testing for 3-MeO-PCP metabolites in blood and urine can confirm the nature of the intoxication by being more specific but do not increase the detection window.

## Conclusion

NPS are a growing public health problem because of the risks associated with their effects, their composition and their pharmacodynamics, which are often unknown.

In this fatal intoxication case, 3-MeO-PCP was identified and quantified in femoral blood and urine. The measured blood concentration of 3-MeO-PCP was the highest concentration ever described in the literature.

For the first time, six metabolites were identified in both human blood and urine. However, the ratio between metabolites and the parent drug were too low to be useful in increasing the detection window of 3-MeO-PCP but represents a further proof of drug administration and may allow the exclusion of any type of contamination or false positive. The analysis of further cases by the scientific community will allow collecting more information that could clarify whether testing for metabolites could be useful in tracing exposure to 3-MeO-PCP when the parent drug is absent.

## Authors’ contributions

Nadia Arbouche contributed in the analysis and wrote the manuscript. Pascal Kintz participated in the analysis, helped to interpret the results and reviewed the manuscript. Cécile Zagdoun collected the biological samples. Laurie Gheddar participated in the analysis. Jean-Sébastien Raul reviewed the manuscript. Alice Ameline coordinated the project, participated in the analysis and reviewed the manuscript. All the authors contributed to the final text and approved it.

## References

[1] UNODC Early Warning Advisory on New Psychoactive Substances. United Nations Office of Drugs and Crime (UNODC); [cited 2020 Dec 9]. Available from: https://www.unodc.org/LSS/Page/NPS

[2] European Monitoring Centre for Drugs and Drug Addiction (EMCDDA). Annual report 2012: the state of the drugs problem in Europe. Luxembourg: Publications Office of the European Union; 2012. [cited 2020 Dec 7]. Available from: http://www.emcdda.europa.eu/attachements.cfm/att_190854_EN_TDAC12001ENC_pdf

[3] Mitchell-Mata C, Thomas B, Peterson B, et al. Two fatal intoxications involving 3-methoxyphencyclidine. J Anal Toxicol. 2017;41:503–507. 28830118 10.1093/jat/bkx048

[4] Morris H, Wallach J. From PCP to MXE: a comprehensive review of the non-medical use of dissociative drugs. Drug Test Anal. 2014;6:614–632. 24678061 10.1002/dta.1620

[5] Mitsuoka T, Hanamura K, Koganezawa N, et al. Assessment of NMDA receptor inhibition of phencyclidine analogues using a high-troughputdrebrin immunocytochemical assay. J Pharmacol Toxicol Methods. 2019;99:106583. 31082488 10.1016/j.vascn.2019.106583

[6] Michely JAA, Manier KM, Caspar AT, et al. New psychoactive substances 3-methoxyphencyclidine (3-MeO-PCP) and 3-methoxyrdicyclidine (3-MeO-PCPy): metabolic fate elucidated with rat urine and human liver preparations and their detectability in urine by GC-MS, “LC-(high resolution)-MSn” and “LC-(high resolution)-MS/MS. Curr Neuropharmacol. 2017;15:692–712. 27758707 10.2174/1570159X14666161018151716 PMC5771046

[7] Ameline A, Greney H, Monassier L, et al. Metabolites to parent 3-MeO-PCP ratio in human urine collec­ted in two fatal cases. J Anal Toxicol. 2019;43:321–324. 30476158 10.1093/jat/bky097

[8] De Jong LAA, Olyslager EJH, Duijst WLJM. The risk of emerging new psychoactive substances: the first fatal 3-MeO-PCP intoxication in The Netherlands. J Forensic Leg Med. 2019;65:101–104. 31129558 10.1016/j.jflm.2019.05.011

[9] Bakota E, Arndt C, Romoser AA, et al. Fatal ­intoxication involving 3-MeO-PCP: a case report and vali­dated method. J Anal Toxicol. 2016;40:504–510. 27339479 10.1093/jat/bkw056

[10] Johansson A, Lindstedt D, Roman M, et al. A non-fatal intoxication and seven deaths involving the dissociative drug 3-MeO-PCP. Forensic Sci Int. 2017;275:76–82. 28324770 10.1016/j.forsciint.2017.02.034

[11] Bertol E, Pascali J, Palumbo D, et al. 3-MeO-PCP intoxication in two young men: first *in vivo* detection in Italy. Forensic Sci Int. 2017;274:7–12. 28057371 10.1016/j.forsciint.2016.12.028

[12] Zidkova M, Hlozek T, Balik M, et al. Two cases of non-fatal intoxication with a novel street hallucinogen: 3-methoxy-phencyclidine. J Anal Toxicol. 2017;41:350–354. 28158698 10.1093/jat/bkx009

[13] Backberg M, Beck O, Helander A. Phencyclidine analog use in Sweden—intoxication cases involving 3-MeO-PCP and 4-MeO-PCP from the STRIDA project. Clin Toxicol. 2015;53:856–864. 10.3109/15563650.2015.1079325 26295489

